# Novel Regeneration Approach for Creating Reusable FO-SPR Probes with NTA Surface Chemistry

**DOI:** 10.3390/nano11010186

**Published:** 2021-01-13

**Authors:** Jia-Huan Qu, Karen Leirs, Remei Escudero, Žiga Strmšek, Roman Jerala, Dragana Spasic, Jeroen Lammertyn

**Affiliations:** 1 Biosensors Group, Department of Biosystems, KU Leuven, Willem de Croylaan 42, 3001 Leuven, Belgium; jiahuan.qu@kuleuven.be (J.-H.Q.); karen.leirs@kuleuven.be (K.L.); remei.escuderofranch@student.kuleuven.be (R.E.); 2 Department of Synthetic Biology and Immunology, National Institute of Chemistry, 1000 Ljubljana, Slovenia; Ziga.Strmsek@ki.si (Ž.S.); roman.jerala@KI.si (R.J.)

**Keywords:** NTA chemistry, surface regeneration, fiber-optic-surface plasmon resonance (FO-SPR), biosensor, His-tag, antibody fragment, SARS-CoV-2 receptor-binding domain (RBD), red fluorescent protein (RFP), protein origami (Tet12SN-RRRR)

## Abstract

To date, surface plasmon resonance (SPR) biosensors have been exploited in numerous different contexts while continuously pushing boundaries in terms of improved sensitivity, specificity, portability and reusability. The latter has attracted attention as a viable alternative to disposable biosensors, also offering prospects for rapid screening of biomolecules or biomolecular interactions. In this context here, we developed an approach to successfully regenerate a fiber-optic (FO)-SPR surface when utilizing cobalt (II)-nitrilotriacetic acid (NTA) surface chemistry. To achieve this, we tested multiple regeneration conditions that can disrupt the NTA chelate on a surface fully saturated with His_6_-tagged antibody fragments (scFv-33H1F7) over ten regeneration cycles. The best surface regeneration was obtained when combining 100 mM EDTA, 500 mM imidazole and 0.5% SDS at pH 8.0 for 1 min with shaking at 150 rpm followed by washing with 0.5 M NaOH for 3 min. The true versatility of the established approach was proven by regenerating the NTA surface for ten cycles with three other model system bioreceptors, different in their size and structure: His_6_-tagged SARS-CoV-2 spike fragment (receptor binding domain, RBD), a red fluorescent protein (RFP) and protein origami carrying 4 RFPs (Tet12SN-RRRR). Enabling the removal of His_6_-tagged bioreceptors from NTA surfaces in a fast and cost-effective manner can have broad applications, spanning from the development of biosensors and various biopharmaceutical analyses to the synthesis of novel biomaterials.

## 1. Introduction

Surface plasmon resonance (SPR) technology has been used for biosensing in various fields, spanning from the pharmaceutical analysis [1,2], medical and food diagnostics [3,4,5,6], to environmental monitoring [7]. In addition to pure analyte quantification, SPR has become a powerful tool to determine binding specificity, affinity and kinetics, thanks to the ability to perform real-time label-free monitoring of biomolecular interactions in both buffer and complex matrices [8,9,10,11]. To date, plenty of effort has been made to improve sensitivity [12], specificity [13], portability [14] and reusability of the SPR sensing surface [15]. The latter has been considered as one of the important strategies to bring down the cost of biosensor systems, especially relevant when it is not feasible to make a disposable sensor, for instance, if high-grade transducers are used or when chip-to-chip variance becomes a source of error [16]. In addition, achieving surface regeneration without losing the sensing performance offers great prospects for rapid screening of biomolecules or biomolecular interactions [17,18,19,20] with high applicability in (1) the development of biosensors [1], (2) various biopharmaceutical analyses and characterization [21], including development of biologics [22], (3) synthesis of novel biomaterials [23], etc. To date, different regeneration strategies have been reported, with chemical regeneration being by far the most widely used [16]. Appropriate regeneration buffers, mediated by low or high pH [24,25] and/or supplemented with detergents [26], chaotropic [27] or competitive [18] agents, have been studied and optimized to either disrupt biomolecular interactions, causing minimal irreversible damage of the molecules immobilized on the sensing surface, or to completely remove the biomolecules from the surface. Other strategies, including thermal and electrochemical regeneration, have also been reported, but overall have been used less, being mainly limited to nucleic acid-based biosensors and electrochemical sensors. In any case, the regeneration approach needs to be chosen carefully, considering the compatibility with the applied surface chemistry and selection of the bioreceptors.

**Figure 1:**
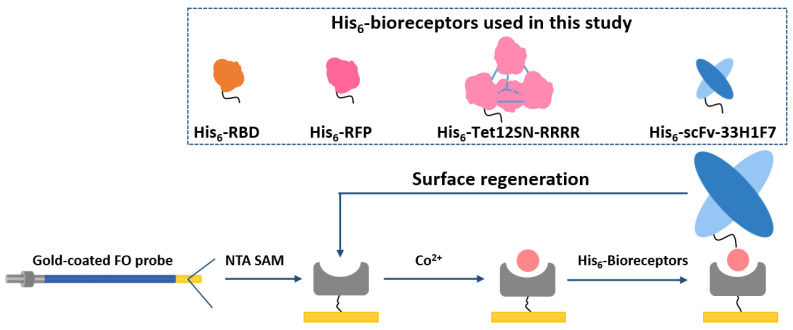
Schematic concept for the regeneration of cobalt (II)-nitrilotriacetic acid (NTA) surface on a gold-coated fiber-optic (FO) sensor probe by reversible removal/immobilization of His_6_-tagged bioreceptors. Four different model systems have been tested to verify the concept, including His_6_-tagged scFv-33H1F7, receptor-binding domain (RBD), red fluorescent protein (RFP) and Tet12SN-RRRR. scFv-33H1F7 has been depicted in the regeneration cycle as one of the examples.

Recently, we have successfully implemented Co(III)-NTA (stands for cobalt (III)-nitrilotriacetic acid) surface chemistry on a fiber-optic (FO)-SPR platform for oriented immobilization of His_6_-tagged bioreceptors [28]. Based on this, we have developed label-free and sandwich immunoassays both in the buffer and complex matrices (i.e., 20- and 10-fold diluted human plasma), with label-free immunoassays being significantly more sensitive compared to our previously developed immunoassays relying on EDC/NHS chemistry [29]. This has opened up the potential for developing a surface regeneration approach and, as such, creating a reusable FO-SPR sensing surface. To achieve this, we use here for the first time Co(II)-NTA chemistry on the FO sensor probe surface, instead of Co(III)-NTA from our previous work. This preference for Co(II)-NTA chemistry originates from the fact that, compared to Co(II), the Co(III) complex exhibits substantially higher association and lower dissociation rate constants (about 20 and 12 orders of magnitudes, respectively), thereby creating a more stable and inert surface, which is unsuitable for disruption and surface regeneration [28]. Contrary to this, without the oxidation step of Co(II) to Co(III), His_6_-tagged proteins can be immobilized on a surface via a coordinate covalent bond with weak affinity. In this context, we first test seven different regeneration conditions for the complete removal of His_6_-tagged antibody fragment (scFv-33H1F7) from the NTA surface. Next, we further optimize the protocol to enable the regeneration of a fully saturated surface across ten regeneration cycles. Finally, we evaluate the applicability of this protocol for regenerating the FO sensor probe surface-functionalized with three different His_6_-tagged proteins, i.e., spike antigen fragment (receptor binding domain, RBD) from severe acute respiratory syndrome coronavirus 2 (SARS-CoV-2), a red fluorescent protein (RFP) and protein origami carrying four RFPs (Tet12SN-RRRR). The applied surface regeneration concept is illustrated in Figure 1.

## 2. Materials and Methods

### 2.1. Reagents and Buffers

Trizma base, phosphate-buffered saline (PBS), ethylenediaminetetraacetic acid (EDTA), disodium salt, dihydrate, imidazole, urea, and sodium dodecyl sulfate (SDS) were purchased from Sigma-Aldrich (Diegem, Belgium). Co(II) chloride hexahydrate was bought from Acros Organics (Geel, Belgium). Sodium chloride, glycine and ethanol were procured from Thermo Fisher Scientific (Erembodegem, Belgium). NTA self-assembling monolayer (SAM) formation reagent (N475-10) was obtained from Dojindo Laboratories (Kumamoto, Japan). Human-derived PAI-1 and anti-PAI-1 His_6_-tagged single-chain variable fragment of mAb MA-33H1F7 (scFv-33H1F7) were produced in the Laboratory for Therapeutic and Diagnostic Antibodies (KU Leuven, Belgium) as previously described [28,30,31]. Recombinant SARS-CoV-2 Spike RBD with His_6_-tag (40,592-V08H), produced in HEK293 cells, was purchased from Sino Biological (Beijing, China). RFP and Tet12SN-RRRR were produced in the lab of Prof. Roman Jerala (National Institute of Chemistry, Ljubljana, Slovenia) with detailed information about production and characterization by Size-exclusion chromatography coupled to multi-angle light scattering (SEC-MALS) in Supplementary information [32]. The following buffers have been prepared for this work: (1) immobilization buffer TBS (50 mM Tris-HCl, 300 mM NaCl, pH 8.0, prepared at a controlled temperature of 25 °C), and (2) regeneration buffers as detailed in Section 2.3. All the buffer solutions were prepared with ultrapure water obtained from a Millipore Synergy UV water purifying system (Millipore SAS, Molsheim, France).

### 2.2. FO-SPR Platform and Preparation of the Gold-Coated FO Sensor Probes

Detailed information on the FO-SPR biosensor commercialized by FOx Biosystems (Diepenbeek, Belgium), which was developed based on our in-house developed FO-SPR biosensor prototype [29], can be found in our previous publication [28] and in Supplementary information (Figure S1). The manufacturing process of the gold-coated FO sensor probe was elaborated previously [33,34] and in Supplementary information.

### 2.3. Testing Different Regeneration Conditions for Removing His_6_-Tagged scFv-33H1F7 Bioreceptors

**Figure 2:**
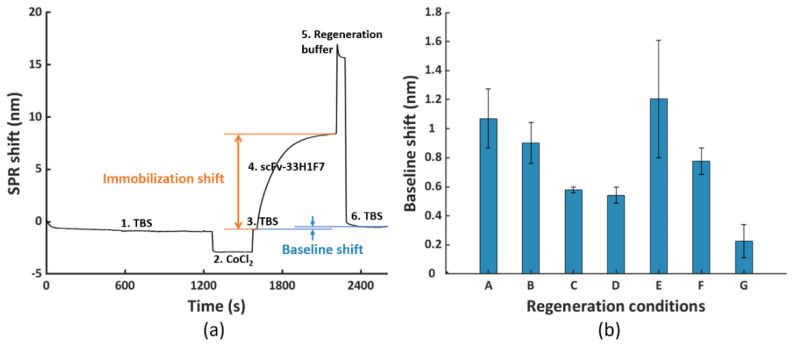
(**a**) The fiber−optic−surface plasmon resonance (FO−SPR) sensorgram representing one regeneration cycle including (1, 3 and 6) immobilization buffer (TBS) stabilization, (2) formation of the Co(II)−NTA chelate, (4) immobilization of His_6_−tagged scFv−33H1F7 (at 20 μg/mL) and (5) removal of scFv−33H1F7 to regenerate the NTA surface following different regeneration conditions. The represented sensorgram was obtained using condition G. (**b**) The obtained baseline shifts after removal of scFv−33H1F7 following seven different regeneration conditions (conditions A to G, as detailed in Section 2.3). Each bar represents the average value of two parallel measurements performed on two different FO sensor probes, with error bars being standard deviations (n_s_ = 2).

First, we functionalized the FO sensor probes with His_6_-tagged bioreceptors on the NTA surface, as presented by the sensorgram in Figure 2a, resembling the process in our previous publication [28]. The gold-coated FO sensor probes were fully immersed in 0.2 mM NTA SAM solution for overnight incubation at 4 °C to form an NTA-coated SAM via thiol bonds. Prior to use, the SAM-coated FO sensor probes were rinsed in ethanol and then transferred to TBS for stabilization. Next, the Co(II)-NTA chelate was formulated by immersing the probe for 5 min in 100 mM CoCl_2_ solution, dissolved by ultrapure water, followed by washing with TBS for 30 s. Subsequently, the activated probe was transferred to the solution of His_6_-tagged scFv-33H1F7 bioreceptor (20 μg/mL, diluted in TBS) for 10 min. Then, the immobilized His_6_-tagged bioreceptors were regenerated by immersing the FO sensor probe in the regeneration buffer. Seven different regeneration conditions were tested, with shaking at 150 rpm for each step and with the FO sensor probe being immersed for 1 min in each of the steps: condition A: 100 mM EDTA, pH 8.0; condition B: 100 mM EDTA, 6 M urea, pH 8.0; condition C: 100 mM EDTA, 500 mM imidazole, pH 8.0; condition D: 100 mM EDTA, 0.5% SDS, pH 8.0; condition E: 10 mM glycine, pH 2.0 (step 1), followed by 100 mM EDTA, 500 mM imidazole, pH 8.0 (step 2); condition F: 0.5% SDS (step 1), followed by 100 mM EDTA, 500 mM imidazole, pH 8.0 (step 2) and condition G: 100 mM EDTA, 500 mM imidazole, 0.5% SDS, pH 8.0. After regeneration, the FO sensor probe was placed back into TBS for 5 min stabilization.

### 2.4. Optimizing the Regeneration Condition for Removing His_6_-Tagged scFv-33H1F7 Bioreceptors

Based on the results from testing different regeneration protocols (see Section 2.3), condition G was selected as the most promising one. Next, to further improve the regeneration performance, extra washing steps were introduced immediately after incubation in regeneration buffer (i.e., before starting the next regeneration cycle). In this context, different washing conditions were tested: 0.1 M NaOH for 5 min with or without shaking at 150 rpm in a first instance, followed by testing three additional conditions with 0.5 M NaOH for 1, 3 or 5 min without shaking. The washing steps were followed by 5 min TBS stabilization. As a control, FO sensor probes were used without extra washing steps, but only with TBS stabilization for 5 min. All the above washing conditions were tested in a total of ten regeneration cycles with 20 μg/mL of scFv-33H1F7 immobilized in each cycle.

### 2.5. Testing the Selected Regeneration Condition with Other His_6_-Tagged Bioreceptors

Next, the selected condition G, followed by washing with 0.5 M NaOH for 3 min, was further tested using other His_6_-tagged proteins, being RBD, RFP and Tet12SN-RRRR. For a total of ten regeneration cycles, 20 μg/mL of each of these proteins was immobilized on the FO sensor probe in each cycle.

### 2.6. Data Analysis

The FO-SPR data were recorded and collected by FOx Biosystems software. Coefficients of variation (CV) were calculated by dividing the mean values by the standard deviations in Microsoft Excel. All the graphs throughout the paper were plotted using programmed scripts in Matlab (The Mathworks, Inc., Natick, MA, USA).

## 3. Results

### 3.1. Testing Different Regeneration Conditions for Removing His_6_-Tagged scFv-33H1F7 Bioreceptors

To develop a protocol for complete regeneration of the FO sensor probe surface, we tested seven different regeneration conditions (i.e., condition A to G, as detailed in Section 2.3). The selection of different buffer components was based on previous work. For instance, 6 M urea, 0.5% SDS, or 10 mM glycine at pH 2.0 are all known for their capacity to denature proteins and accelerate the regeneration process [16]. EDTA and imidazole have been previously described as competitive reagents of NTA and His-tag, respectively [20], and as such have been widely used during protein purification with NTA columns for column recharging and protein elution, respectively [35]. In addition, EDTA has been included here in different buffer combinations since it is a stronger chelator than NTA, thus being essential to disrupt the chelate of NTA-Co(II)-His_6_-tagged protein [36].

For testing these seven different conditions, we used His_6_-tagged scFv-33H1F7 since an antibody (fragment) represents one of the most classical model systems for bioreceptors. This bioreceptor was first immobilized on the FO sensor probe at 20 μg/mL, as explained in Section 2.3, followed by surface regeneration, which together constituted one regeneration cycle (illustrated with the sensorgram in Figure 2a). This way, we obtained seven different sensorgrams for seven different conditions, with a representative one depicted for condition G in Figure 2a. Based on this, we calculated baseline shifts by subtracting the TBS baseline value before bioreceptor immobilization from that after surface regeneration within the cycle (Figure 2b). These baseline shifts served to evaluate the efficiency of different regeneration conditions, with a value close to zero implying a complete removal of His_6_-tagged proteins and a sufficient surface regeneration. Compared to condition A that had only EDTA, adding urea (condition B) did not have any additional influence on the regeneration efficiency, whereas adding imidazole or SDS improved the regeneration by bringing down the baseline shift (condition C and condition D, respectively; Figure 2b). Furthermore, we also tested the influence of a lower pH value by introducing 10 mM glycine (i.e., pH 2.0) prior to using the combination of EDTA and imidazole because the protonation of the imidazole ring in His-tag occurs below pH 6.0 [37]. However, lower pH did not provide any additional benefits in removing the His_6_-tagged proteins, as observed from the high baseline shift (condition E), which may be due to the adsorption between the positively charged proteins and slightly negatively charged NTA surface at low pH value. Next, we attempted to combine SDS with EDTA and imidazole by either applying it stepwise (condition F) or combined together in one solution (condition G). Overall, condition G (100 mM EDTA, 500 mM imidazole, 0.5% SDS at pH 8.0 for 1 min with shaking at 150 rpm) resulted in the lowest baseline shift (i.e., 0.23 nm) out of the seven tested conditions. Although for some of the tested conditions (e.g., condition A, B and E) the variability was larger than for the others, the difference between the baseline shift for condition G and all the other tested conditions was prominent enough to select this condition for further optimization in the subsequent experiments.

### 3.2. Optimization of the Regeneration Condition for Removing His_6_-Tagged scFv-33H1F7 Bioreceptors

**Figure 3:**
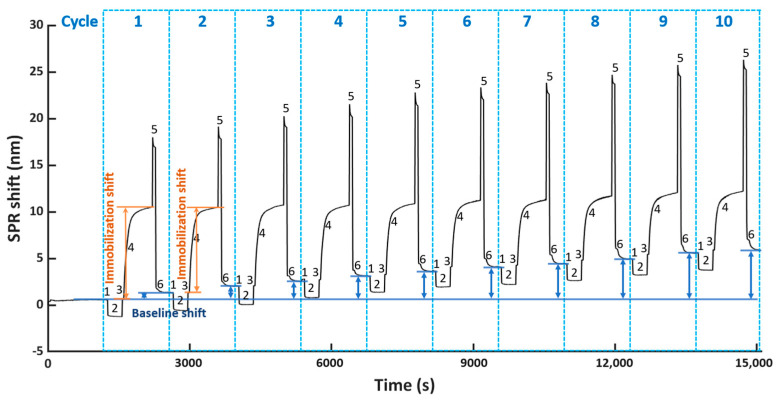
The FO−SPR sensorgram representing ten regeneration cycles, including (1, 3 and 6) TBS stabilization, (2) formation of the Co(II)−NTA chelate, (4) immobilization of scFv−33H1F7 at 20 μg/mL and (5) removal of scFv−33H1F7 to regenerate the NTA surface using condition G. The baseline shift (blue arrows) for each cycle and the immobilization shift for cycle 1 and 2 (orange arrow) are depicted.

To evaluate the reusability of the NTA surface, we performed ten regeneration cycles on a single FO sensor probe, where each regeneration cycle consists of bioreceptor immobilization, followed by its removal. Here, we used the selected condition G for regeneration, whereas His_6_-tagged scFv-33H1F7 bioreceptor was immobilized at 20 μg/mL for 10 min. This concentration was chosen because it results in a saturated FO surface, often preferred when developing biosensors to ensure complete surface coverage with bioreceptors, good reproducibility of bioreceptor immobilization and low(er) nonspecific adsorption of molecules from complex matrices onto the biosensing surface [28,29,34] The resulting sensorgram is displayed in Figure 3, where both the baseline and immobilization shifts were monitored over ten different regeneration cycles. The baseline shift for each cycle was calculated similarly as in Figure 2a, by subtracting the TBS baseline of cycle 1 before bioreceptor immobilization from that after surface regeneration in each corresponding cycle (depicted in Figure 3 with a blue arrow for each cycle). The immobilization shift in each cycle was determined based on the obtained SPR shift, as indicated in Figure 2a (i.e., by subtracting the mean of the first twenty points from the mean of the last twenty during immobilization, depicted in Figure 3 as step 4 of the sensorgram).

**Figure 4:**
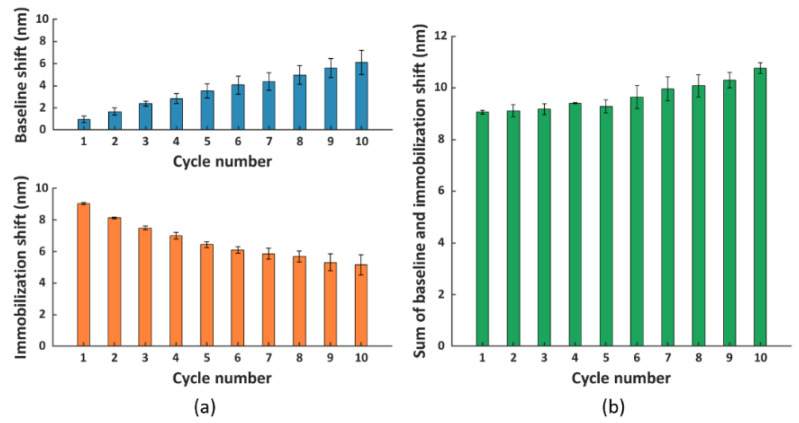
(**a**) The baseline (blue) and immobilization (orange) shift obtained in different cycles. (**b**) The sum of the immobilization shift per cycle and the baseline shift from the preceding cycle (except for cycle 1, where only immobilization shift is depicted as there is no baseline shift prior to this cycle). Each bar represents the average value of two parallel measurements performed on two different FO sensor probes, with error bars being standard deviations of the overall shift (n_s_ = 2).

Based on Figure 4a, we observed an ascending trend of baseline shifts (top panel) and a descending trend of immobilization shifts (bottom panel) compared to cycle 1. The increase in baseline shift implied an accumulation of proteins on the surface due to incomplete surface regeneration, which probably hindered the immobilization of scFv-33H1F7 in the next cycles, resulting in a decrease in immobilization shift. Even though we observed such changes in both the baseline and immobilization shifts after several cycles, surprisingly, the overall shift (i.e., the sum of the immobilization shift per cycle and the baseline shift from the preceding cycle) did not demonstrate statistically significant difference across the first eight cycles (Figure 4b). This suggested that the total amount of immobilized bioreceptors on the FO sensor probe surface was largely constant throughout these regeneration cycles.

**Figure 5:**
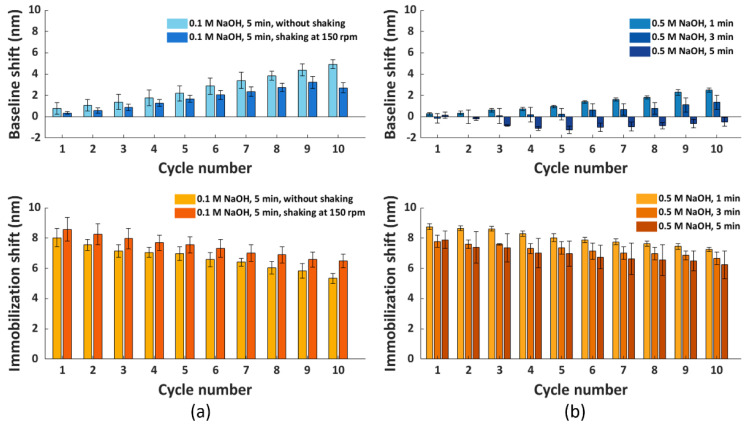
The baseline and immobilization shifts obtained for ten regeneration cycles, with His_6_−tagged scFv−33H1F7 immobilized at 20 μg/mL in each cycle. The FO sensor probe surface regeneration was performed using condition G, combined with different washing conditions: (**a**) 0.1 M NaOH for 5 min with or without shaking at 150 rpm and (**b**) 0.5 M NaOH for 1, 3 or 5 min without shaking. Each bar represents the average value from two parallel measurements performed on two different FO sensor probes, with error bars being standard deviations (n_s_ = 2).

Nevertheless, based on these results, we concluded that the regeneration protocol required further optimization to achieve complete removal of proteins from the surface. Therefore, we included an extra washing step after the incubation in a regeneration buffer. Since the NTA surface exhibits a negative charge after the disruption of the NTA chelate, we used washing buffers with a high pH to impose a negative charge on the proteins removed from the NTA surface, thereby creating a repelling force between them [37]. In this context, we first tested 5 min surface washing using 0.1 M NaOH (pH 13) either with or without shaking at 150 rpm, and we applied it immediately after the regeneration buffer step [35]. To evaluate if there was an improvement in surface regeneration compared to condition G, the baseline and immobilization shifts were calculated over a total of ten regeneration cycles (Figure 5a). Similarly, as in the previous experiments, His_6_-tagged scFv-33H1F7 was immobilized at 20 μg/mL in each cycle to achieve complete surface saturation. Compared to Figure 4a, the baseline shifts were generally lower, and there was a less pronounced difference in immobilization shift over different cycles when performing the extra washing step, suggesting some improvements compared to the original condition G. However, even with these improvements, the increasing trend in the baseline shift was still obvious, accompanied with a decreasing trend in the immobilization shift (Figure 5a) using both washing conditions (also depicted in Figure S2a,b), indicating an incomplete surface regeneration.

**Figure 6:**
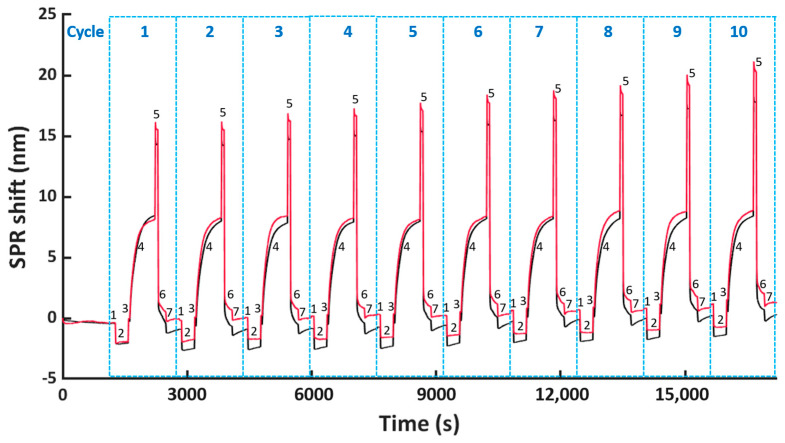
The FO-SPR sensorgram obtained from two parallel FO probes representing ten regeneration cycles of His_6_−tagged scFv−33H1F7, including the following steps: (1, 3 and 7) TBS stabilization, (2) formation of the Co(II)−NTA chelate, (4) immobilization of scFv−33H1F7 at 20 μg/mL, (5) removal of scFv−33H1F7 and (6) washing with NaOH, using the optimized regeneration protocol (i.e., condition G combined with 3 min washing in 0.5 M NaOH).

Therefore, we decided to further improve the washing of the FO sensor probe surface by increasing the concentration of NaOH to 0.5 M. Here; we tested 5 min of washing, as well as shorter periods of 1 and 3 min to reduce the harsh conditions associated with the high NaOH concentration. Previously tested shaking during the washing step (Figure 5a) was not included here since shaking did not lead to any significant improvements within the first nine regeneration cycles when comparing light and dark blue bars in Figure 5a, top panel. Similarly, as before, ten regeneration cycles of His_6_-tagged scFv-33H1F7 were performed, with the bioreceptor being immobilized at 20 μg/mL in each cycle. According to the baseline and immobilization shifts (Figure 5b), we selected a 0.5 M NaOH washing step with 3 min duration to be combined with condition G because it resulted in the best overall performance among three tested conditions with (1) less than 1.5 nm variation of baseline shifts across all ten cycles (Figure S2c) and (2) an overall 14.5% decrease in the immobilization shifts between cycle 1 and cycle 10 with a CV of 4.9% (Figure S2d). A full sensorgram obtained with this protocol is depicted in Figure 6 and was defined as the final regeneration protocol for all the following experiments.

### 3.3. Validation of the Selected Regeneration Condition by Implementing Other His_6_-Tagged Bioreceptors

**Figure 7:**
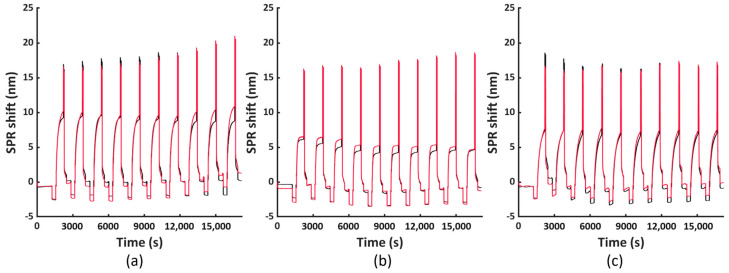
The FO−SPR sensorgrams obtained from two parallel FO probes representing ten regeneration cycles, performed using the optimized regeneration protocol (i.e., condition G combined with 3 min washing in 0.5 M NaOH), shown for different His_6_-tagged bioreceptor model systems: (**a**) RBD, (**b**) RFP and (**c**) Tet12SN−RRRR.

**Figure 8:**
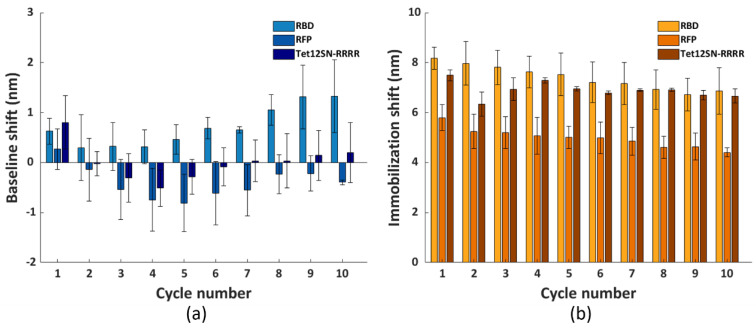
The (**a**) baseline and (**b**) immobilization shifts for each cycle, obtained with immobilizing RBD, RFP and Tet12SN−RRRR at 20 μg/mL. The surface regeneration was performed using condition G and washing with 0.5 M NaOH for 3 min, for a total of ten cycles. Each bar represents the average value from two parallel measurements performed on two different FO sensor probes, with error bars being standard deviations (n_s_ = 2).

To assess the versatility of the selected regeneration condition, we conducted the same regeneration process using three other model systems, namely His_6_-tagged RBD, RFP and Tet12SN-RRRR. These three proteins represent another type of bioreceptors, differing in their structure and size (i.e., molecular weight (Mw) being 26.5 kDa, 26.7 kDa and 154.7 kDa, respectively) compared to the antibody fragment scFv-33H1F7 (Mw: 37.9 kDa) used for the optimization of the regeneration protocol. The ten regeneration cycles were performed similarly as above, with RBD, RFP or Tet12SN-RRRR being immobilized at 20 μg/mL in each cycle to reach complete surface saturation and applying condition G combined with the 3 min washing step in 0.5 M NaOH for their removal. The recorded sensorgrams are displayed in Figure 7a–c. This, together with the calculated baseline or immobilization shifts across all the cycles (Figure 8), revealed that (1) the maximal baseline shift was just over 1 nm (i.e., for RBD) while being much lower for all the other model systems (Figure S3a), whereas (2) the decrease in the immobilization shifts between cycle 1 and cycle 10 was 10.9%, 16.1% and 24% for Tet12SN-RRRR, RBD and RFP, respectively (Figure S3b). This was largely comparable to the results obtained above with the antibody fragment, proving that the developed regeneration condition is very robust as it was directly implemented for the removal of different types of His_6_-tagged proteins without any additional optimization, revealing its great potential for more extensive use in different applications. However, it is important to note that, based on a slightly different trend of baseline shifts for these three model proteins, one could decide to additionally fine-tune the same regeneration protocol when working with various bioreceptors.

## 4. Discussion

In this paper, we developed a regeneration approach for removing different His_6_-tagged bioreceptors from the NTA surface that, as such, enables the reusability of FO sensing probes. To achieve this, we used here for the first time Co(II)-NTA chemistry on the FO sensor probe surface, rather than Co(III)-NTA established in our previous work [28]. Skipping the oxidation step of Co(II) to Co(III) was essential to promote a coordinate covalent bond of His_6_-tagged proteins to the surface with weak affinity [38].

In this context, we first tested seven different regeneration conditions for disrupting the NTA chelate on the FO sensor probes functionalized with an antibody fragment (His_6_-tagged scFv-33H1F7) as a model system. These seven conditions have combined, in varying ways, the reagents competing with NTA (like EDTA) and His-tag (like imidazole), as well as protein denaturants (i.e., urea, SDS, and glycine at pH 2.0), whose selection was based on previous work [16,20,35,36]. The best surface regeneration was obtained when combining 100 mM EDTA, 500 mM imidazole and 0.5% SDS at pH 8.0 for 1 min with shaking at 150 rpm, which was evaluated based on the smallest baseline shift between the beginning and the end of the regeneration cycle. This protocol was further fine-tuned towards robust regeneration across ten regeneration cycles by adding an extra washing step for 3 min in NaOH buffer at pH above 13. The high pH value of the washing buffer significantly improved the completeness of bioreceptor removal from the surface, most probably by causing a negative charge on the proteins removed from the sensor, thereby creating a repelling force against the negative NTA surface. Notably, this regeneration protocol was established for a surface completely saturated with bioreceptors, a condition often selected in biosensing as it assures complete surface coverage with bioreceptors, good reproducibility in bioreceptor immobilization, while minimizing biofouling of the biosensor when introduced in complex matrices [28,29,34]. It is also important to notice that, although this final protocol included some elements of the protocols extensively used for protein purification, it achieved a much faster regeneration of the NTA surface compared with usually long and multistep recharging of NTA columns during protein purification [35].

Importantly, the selected regeneration condition was validated using three additional model systems, being His_6_-tagged RBD, RFP and Tet12SN-RRRR, differing among each other and from scFv-33H1F7 in their size and structure. Similar to the scFv-33H1F7 model system, almost complete regeneration was achieved without any significant impact on the baseline or immobilization shifts across ten cycles while having a completely saturated surface in each cycle. This demonstrated the versatility of the established regeneration approach. Unlike most of the previous work [20,39], this study also included the recorded complete sensorgrams of all the used model systems, providing full insight into FO-SPR surface response for ten regeneration cycles.

In conclusion, this work presented the development of a robust regeneration protocol for making NTA-based FO sensor probes reusable. Such surfaces can find their applications for the rapid screening of reagents, like antibodies or drugs, which could reduce both the time and cost of the analysis. Additionally, this regeneration approach is not limited to the FO-SPR sensing platform but can also be applied to other platforms using NTA chemistry to immobilize His_6_-tagged reagents. This surface chemistry also shows advantages over other strategies for oriented immobilization of bioreceptors, such as biotin-streptavidin or protein A/G [40]: (1) because NTA is extensively used for protein purification, many proteins are produced with a His_6_-tag [41], making them directly amenable for immobilization through NTA surface chemistry as opposed to necessary biotinylation step for binding to streptavidin and (2) small His_6_-tag directly bound to the NTA assures molecular interactions closer to the biosensing surface compared to streptavidin-biotin and protein A/G immobilization approaches, which is essential when working with SPR sensors for instance [42]. Finally, due to the flexibility of using Co(II)- or Co(III)-NTA surface chemistry, important follow-up work is foreseen to explore the feasibility of surface regeneration when the analyte is bound to the bioreceptor, for example, by removing the complex of His_6_-tagged bioreceptor and analyte together (with Co(II)), or even removing only the analyte from the bioreceptor (with Co(III)).

## Data Availability

Data available on request.

## Supplementary Materials

The following are available online at https://www.mdpi.com/2079-4991/11/1/186/s1, Figure S1: The FO-SPR biosensor commercialized by FOx Biosystems; Figure S2: Summary of line charts for the baseline shifts obtained from ten regeneration cycles and the ratios of the immobilization shifts per cycle compared to cycle 1 at different conditions; Figure S3: Summary of line charts for the baseline shifts obtained from ten cycles and the ratios of the immobilization shifts per cycle compared to cycle 1 when immobilizing RBD, RFP and Tet12SN-RRRR with the optimized condition.
