# Supplementary material for: Novel Regeneration Approach for Creating Reusable FO-SPR Probes with NTA Surface Chemistry
Source: Nanomaterials (Basel). 2021 Jan 13;11(1):186. doi: 10.3390/nano11010186 (PMC7828519; doi:10.3390/nano11010186)
Supplement: Supplementary file 1 [file nanomaterials-11-00186-s001.pdf]

nanomaterials

MDPI

Article

Novel Regeneration Approach for Creating Reusable FO-SPR Probes with NTA Surface Chemistry

Jia-Huan Qu [1], Karen Leirs [1], Remei Escudero [1], Žiga Strmšek [2], Roman Jerala [2], Dragana Spasic [1,*] and Jeroen Lammertyn [1,*]

1 Department of Biosystems, Biosensors Group, KU Leuven, Willem de Croylaan 42, 3001 Leuven, Belgium; jiahuan.qu@kuleuven.be (J.-H.Q.); karen.leirs@kuleuven.be (K.L.); remei.escuderofranch@student.kuleuven.be (R.E.)

2 Department of Synthetic Biology and Immunology, National Institute of Chemistry, 1000 Ljubljana, Slovenia; Ziga.Strmsek@ki.si (Ž.S.); roman.jerala@KI.si (R.J.)

* Correspondence: dragana.spasic@kuleuven.be (D.S.); jeroen.lammertyn@kuleuven.be (J.L.); Tel.: +32-16-37-71-18 (D.S.); +32-16-32-14-59 (J.L.)

S1. Materials and Methods

S1.1 Production and characterization of His$_6$-tagged RFP and Tet12SN-RRRR

Molecular cloning

His$_6$-tagged RFP gene was inserted into pET-41a between *NdeI* and *XhoI* sites using classical cloning techniques and Gibson assembly. His$_6$-tagged Tet12SN-RRRR was obtained by Golden Gate Assembly, as described in the previous work [1]. In short, the sequence was divided into nine segments, and each segment was flanked by custom Golden Gate overhangs. Upon digestion with *BsaI*, complementary overhangs would enable hierarcial and scarless assembly into the final construct and it's insertion into the target pET-41a vector between *NdeI* and *XhoI* sites.

Protein Expression and Purification

His$_6$-tagged RFP and Tet12SN-RRRR were prepared according to a previously described protocol [1]. In short, the protein of interest was expressed in *E. coli* NiCO21 (DE3) cells and grown for 4 more hours after the induction with IPTG at 30 °C. Pellet from 3 L of fermentation was collected and stored at -80 °C. Bacterial pellet was resuspended in lysis buffer, cells were lysed with sonication, clarified with centrifugation and soluble fraction was loaded onto gravity columns, loaded with pre-equilibrated NiNTA resin. After extensive washing, the protein of interest was eluted, the fractions of interest were pooled and further polished with a step of size exclusion (HiLoad 26/600 Superdex 200 pg). After size exclusion, fractions of interest were pooled again, concentrated, flash-frozen in liquid nitrogen and stored at -80 °C.

Size-exclusion chromatography coupled to multi-angle light scattering (SEC-MALS)

SEC-MALS experiments were performed on a Waters e2695 HPLC system coupled with a 2489 UV detector (Waters, MA, USA), a Dawn8+ multiple-angle light scattering detector (Wyatt, CA, USA) and refractive index (RI) detector RI500 (Shodex, Japan). Filtered sample (Durapore 0.1 μm centrifuge filters (Merck Millipore, MA, USA)) was injected onto a Superdex 200 increase 10/300 GL column (Cytiva, USA) and eluted in 20 mM Tris at pH 7.5, 150 mM NaCl at 0.5 mL/min. Data was analysed with Astra 7.0 (Wyatt, USA).

S1.2. FO-SPR platform and manufacturing of FO-SPR sensor probes

A multimode optical fiber (TEQS, Thorlabs, Munich, Germany) with a core diameter of 400 μm and a total length of 4.3 cm was used in the experiments. Approximately 0.6 cm of the jacket was detached from one end of the fiber and the cladding was removed with an acetone soaked dust free tissue. The sensing zone was cleaned with ethanol before sputtering with a 50 nm gold layer using a sputter coater (Quorum Q150T ES, Quorum Technologies, East Sussex, UK).

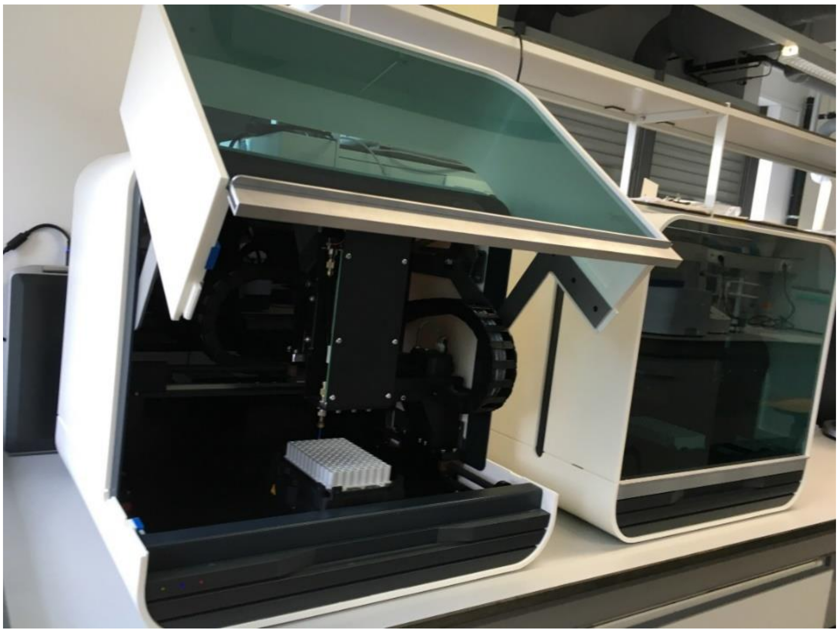

Figure S1. The commercialized FO-SPR biosensor by FOx Biosystems.

S2. Results

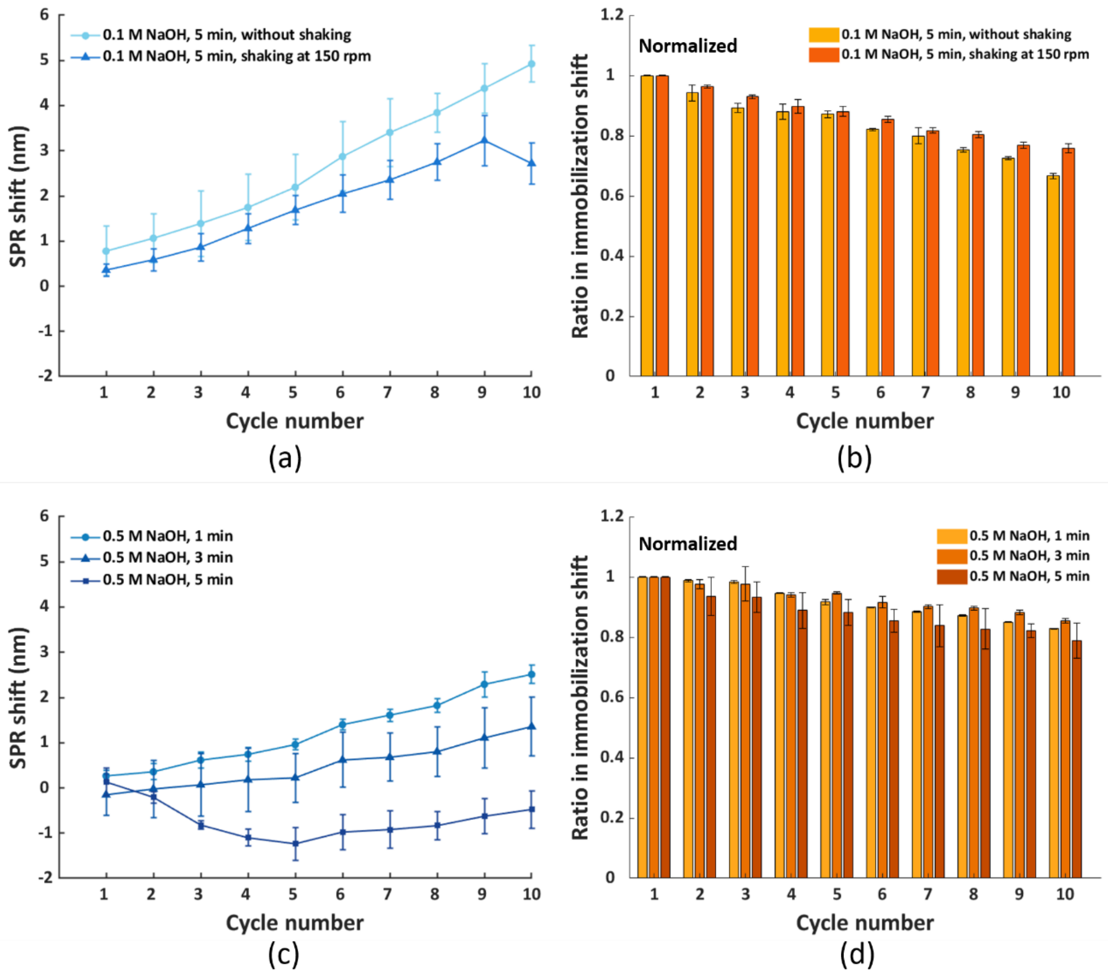

Figure S2. (**a**) and (**c**) Summary of line charts for the baseline shifts obtained from ten regeneration cycles using different conditions (0.1 M NaOH and 0.5 M NaOH, respectively). The lines connecting different points are just guide for the eye. (**b**) and (**d**) The ratios of the immobilization shifts per cycle compared to that in Cycle 1, using different regeneration conditions (0.1 M NaOH and 0.5 M NaOH, respectively). All experiments were performed with scFv-33H1F7 immobilized at 20 μg/mL in each cycle. Error bars represent standard deviations (n_s=2).

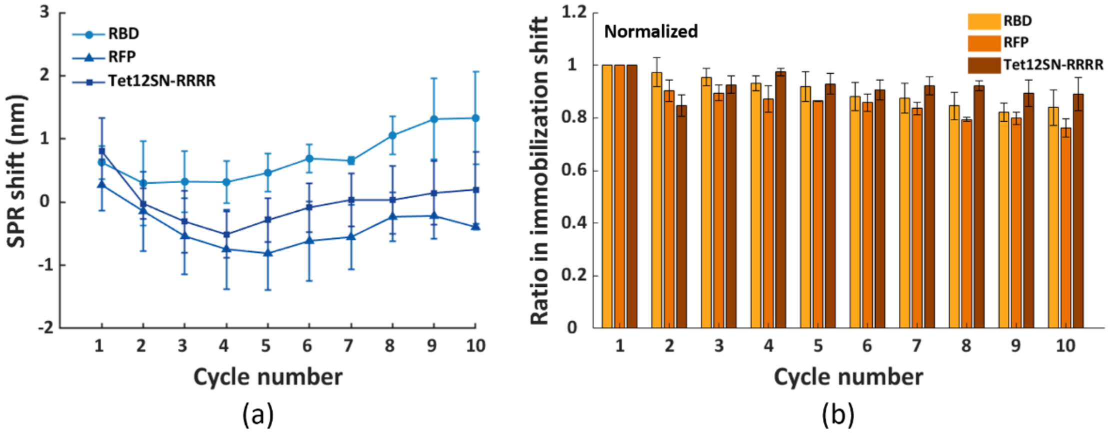

Figure S3. (**a**) Summary of line charts for the baseline shifts obtained from ten regeneration cycles. (**b**)The ratios of the immobilization shift per cycle compared to that in Cycle 1. Both the baseline and immobilization shifts were obtained with immobilizing RBD, RFP and Tet12SN-RRRR. Error bars represent standard deviations (n_s=2).

References

1. Strmšek Žiga, Aupič Jana, Lapenta Fabio, Vidmar Sara, Lainšček Duško, Gradišar Helena, J.R. Coiled-coil origami as a scaffold for presenting protein domains. *Manuscr. Prep.*
